# *Ccr2* deletion dissociates cavity size and tau pathology after mild traumatic brain injury

**DOI:** 10.1186/s12974-015-0443-0

**Published:** 2015-12-03

**Authors:** Stefka Gyoneva, Daniel Kim, Atsuko Katsumoto, O. Nicole Kokiko-Cochran, Bruce T. Lamb, Richard M. Ransohoff

**Affiliations:** Department of Neurosciences, Lerner Research Institute, Cleveland Clinic Foundation, Cleveland, OH USA; Department of Chemistry, Case Western Reserve University, Cleveland, OH USA; Neuroinflammation Research Center, Cleveland Clinic Foundation, Cleveland, OH USA; Neuroimmunology, Biogen, 225 Binney St, Cambridge, MA 02142 USA

**Keywords:** Traumatic brain injury, CCR2, Tau, MAPT, Monocyte

## Abstract

**Background:**

Millions of people experience traumatic brain injury (TBI) as a result of falls, car accidents, sports injury, and blast. TBI has been associated with the development of neurodegenerative conditions such as Alzheimer’s disease (AD) and chronic traumatic encephalopathy (CTE). In the initial hours and days, the pathology of TBI comprises neuronal injury, breakdown of the blood–brain barrier, and inflammation. At the cellular level, the inflammatory reaction consists of responses by brain-resident microglia, astrocytes, and vascular elements as well as infiltration of peripheral cells. After TBI, signaling by chemokine (C-C motif) ligand 2 (CCL2) to the chemokine (C-C motif) receptor 2 (CCR2) is a key regulator of brain infiltration by monocytes.

**Methods:**

We utilized mice with one or both copies of *Ccr2* disrupted by red fluorescent protein (RFP, *Ccr2*^*RFP*/+^ and *Ccr2*^*RFP*/*RFP*^). We subjected these mice to the mild lateral fluid percussion model of TBI and examined several pathological outcomes 3 days later in order to determine the effects of altered monocyte entry into the brain.

**Results:**

*Ccr2* deletion reduced monocyte infiltration, diminished lesion cavity volume, and lessened axonal damage after mild TBI, but the microglial reaction to the lesion was not affected. We further examined phosphorylation of the microtubule-associated protein tau, which aggregates in brains of people with TBI, AD, and CTE. Surprisingly, *Ccr2* deletion was associated with increased tau mislocalization to the cell body in the cortex and hippocampus by tissue staining and increased levels of phosphorylated tau in the hippocampus by Western blot.

**Conclusions:**

Disruption of CCR2 enhanced tau pathology and reduced cavity volume in the context of TBI. The data reveal a complex role for CCR2^+^ monocytes in TBI, as monitored by cavity volume, axonal damage, and tau phosphorylation.

## Background

Traumatic brain injury (TBI) is a common condition in today’s society. It is variable in extent and circumstance and imposes enormous suffering and expense. The injury can be sustained in a single event or in a repetitive fashion, can be either closed head or penetrating, and ranges in severity from mild to severe. About 75 % of TBI is closed head in nature and mild in intensity [1]. Further contributing to patient suffering and impact on society, TBI is linked to the development of neurodegenerative conditions. For example, TBI of varying intensity is associated with increased risk or earlier onset of Alzheimer’s disease (AD), and repetitive mild TBI is associated with chronic traumatic encephalopathy (CTE) [2–8].

One common pathological feature of TBI, AD, and CTE is the hyperphosphorylation and mislocalization of the microtubule-associated protein tau (MAPT, here termed tau) [9]. Tau contains numerous potential phosphorylation sites and is a target of multiple kinases. In physiological conditions, tau is phosphorylated at a few sites and is located in axons where it binds to microtubules to stabilize the axonal cytoskeleton. However, in pathological conditions, tau can be phosphorylated at multiple additional sites. The (hyper)phosphorylated tau (pTau) dissociates from microtubules in the axon, translocates to the cell body and proximal dendrites, and aggregates in structures termed neurofibrillary tangles, leading to impaired axonal function [9]. While the mechanisms driving tau phosphorylation and tangle formation are not well established, there is substantial evidence suggesting that inflammation can promote this process [10–12].

A prominent feature of TBI is the development of an inflammatory reaction within minutes of the injury event [13–17]. Cells at the site of injury secrete cytokines and chemokines that lead to the recruitment of peripheral immune cells—initially neutrophils, which are quickly replaced by monocytes. Concurrently, there is activation of brain-resident astrocytes and microglia. The inflammatory reaction could contribute to the progression of axonal pathology and tissue damage and is thus a potential target to ameliorate TBI pathology [13, 17, 18].

Although inflammation increases tau phosphorylation and pTau is detected in central nervous system (CNS) tissues of TBI patients and in animal models, whether post-injury inflammation affects pTau levels in the context of TBI has not been addressed. Here, we used the lateral fluid percussion injury (LFPI) model of TBI [19, 20] to study how modulation of the monocytic reaction after TBI influences tau phosphorylation. In the context of TBI, the monocytic population is primarily represented by CD45^lo^CX3CR1^hi^CCR2^−^ microglia, CD45^hi^CX3CR1^lo^CCR2^hi^ inflammatory monocytes, and CD45^hi^CX3CR1^hi^CCr2^lo^ patrolling monocytes [21–24], but perivascular and meningeal macrophages may also play a role. We employed mice deficient for either chemokine (C-X3-C motif) receptor 1 (*Cx3cr1*^*GFP*/*GFP*^ mice) or chemokine (C-C motif) receptor 2 (*Ccr2*^*RFP*/*RFP*^ mice). *Ccr2*^*RFP*/*RFP*^ mice display reduced infiltration of Ly6C^hi^ inflammatory monocytes into the brain after TBI [25]. Here, *Cx3cr1*^*GFP*/*GFP*^ mice showed no difference in TBI pathology compared to wild type (WT) mice, but *Ccr2*^*RFP*/*RFP*^ mice had reduced lesion volume and axonal pathology. Surprisingly, *Ccr2*^*RFP*/*RFP*^ mice also exhibited increased levels and mislocalization of pTau in the cortex and hippocampus, suggesting that monocyte-dependent inflammation exerts distinct effects on tissue loss as compared to tau phosphorylation after TBI.

## Methods

### Animals and TBI induction

All procedures performed on animals were reviewed and approved by the Institutional Animal Care and Use Committee of the Cleveland Clinic. We employed two mouse strains (*Cx3cr1*^*GFP*/*GFP*^ and *Ccr2*^*RFP*/*RFP*^) that allowed us to manipulate the cellular reaction after TBI and at the same time visualize the inflammatory cells of interest [26]. *Cx3cr1*^*GFP*/*GFP*^ mice (green fluorescent protein (GFP) expression in microglia and patrolling CCR2^lo^ monocytes) were maintained on the *Ccr2*^*RFP*/+^ background in which only one copy of *Ccr2* is disrupted (red fluorescent protein (RFP) expression in inflammatory CCR2^hi^ monocytes, some T cells). Similarly, for some experiments *Ccr2*^*RFP*/*RFP*^ mice were maintained on the *Cx3cr1*^*GFP*/+^ background. There were no statistical differences in the responses to TBI by *Ccr2*^*RFP*/*RFP*^*; Cx3cr1*^*+/+*^ and *Ccr2*^*RFP*/*RFP*^; *Cx3cr1*^*GFP*/+^ mice (data not shown) and the two genotypes were pooled together for analysis. Microglia were identified by Iba1 staining in *Ccr2*^*RFP*/*RFP*^ mice at 3 days post injury (dpi), before other cell types have upregulated Iba1 expression [27].

To induce TBI, we performed lateral fluid percussion injury as described before [19]. Briefly, 8–10-week-old male and female mice were anesthetized with 100 mg/kg ketamine/10 mg/kg xylazine; the fur on top of the head was shaved, and the skin was cut and moved to the side. A craniotomy with ~3 mm diameter was opened on the right side of the central suture, halfway between Bregma and Lambda, without disturbing the underlying dura mater. A modified Leur-Lok hub was placed around the craniotomy and sealed in place with dental acrylic. The mice were allowed to recover from anesthesia and returned to their home cages. On the next day, the mice were anesthetized again and attached to a fluid percussion device (AmScien Instruments FP-302) by the Leur-Lok hub. The device was calibrated to deliver mild injury with pressure intensities between 0.4 and 0.6 atm. After injury, the hub was removed, the skin was sutured, and mice were returned to their home cages to recover. For all experiments, the mice were euthanized 3 dpi.

### Tissue staining

Tissue staining was used to evaluate the extent of the inflammatory reaction after TBI, lesion volume, axonal pathology, and tau phosphorylation and localization. At 3 dpi, mice were deeply anesthetized with ketamine/xylazine and perfused with ice-cold phosphate-buffered saline (PBS) followed by 4 % paraformaldehyde (PFA) in PBS. The brains were isolated and postfixed in 4 % PFA overnight and sectioned on a sliding microtome at 30 μm thickness. During staining, all washes were performed three times for 5 min each in 0.1 % triton X-100 in PBS. Antibody solutions were prepared in PBS unless otherwise noted.

To visualize the inflammatory reaction after TBI, serial sections spaced 150 μm apart and spanning ~4 mm thickness around the injury cavity were blocked in 10 % normal goat serum (NGS) and stained overnight at 4 °C with mouse anti-GFP (UCDavis/NIH NeuroMab Facility #75-132, 1:8000 dilution), or rabbit anti-Iba1 (Wako #019-19741, 1:1000) antibodies to identify microglia, and rabbit or rat anti-RFP (Abcam #ab62341, 1:1000 and Chromotek #5 F8 α-Red, 1:1000, respectively) antibodies to identify infiltrating monocytes. The secondary antibodies (Goat anti-mouse-Alexa 488, Invitrogen #A11029; anti-Rabbit IgG-Alexa Fluor 488, Invitrogen #A11008; anti-rabbit-Alexa 594, Invitrogen #A11037; anti-Rat IgG-Alexa Fluor 594, Invitrogen #A11007, all at 1:1000 dilution) were applied for 1 h at room temperature (RT). The sections were mounted on large glass slides, coverslipped and imaged.

Axonal pathology was assessed with amyloid precursor protein (APP) staining which accumulates in axonal swellings of damaged neurons [28]. The sections were incubated in 0.3 % H_2_O_2_ for 30 min at RT to inactivate endogenous peroxidases. Antigen retrieval was performed in 1× Target retrieval solution (Dako Cytomation #S1699) containing 0.5 % Tween in PBS at 95 °C for 10–15 min. After blocking in 10 % NGS, primary rabbit anti-APP antibody (Invitrogen #51-2700) was applied overnight at 4 °C and secondary biotinylated goat anti-rabbit antibody (Vector BA-1000) was applied for 1 h at RT. The signal was detected using the ABC Elite Kit (Vector PK-6100) and DAB substrate kit (Vector SK-4100) according to the manufacturer’s instructions. Sections were allowed to dry and coverslipped with hardset mounting medium (Fisher Scientific #SP15).

For phosphorylated tau, after antigen retrieval and blocking in 5 % NGS and 0.3 % Triton X-100 in PBS for 2 h at RT, the localization of pTau was detected with the mouse anti-AT8 antibody (ThermoSci mn1020, 1:500 dilution in 5 % NGS, 0.3 % Triton X-100 in PBS) overnight at 4 °C. The secondary antibody used, goat anti-mouse-Alexa 647 (Invitrogen #A21235, 1:1000 dilution), was chosen not to interfere with the signal of genetically encoded GFP and RFP and was applied for 1 h at RT. The sections were mounted on glass slides and coverslipped with VectaShield with DAPI medium (Vector H-1200).

### Imaging and image quantification

Sections stained with GFP (or Iba1) and RFP were imaged on a Leica CTR5500 microscope equipped with a QImaging Retiga EXi FAST 13941 MONO camera at 10× magnification. Tiling of consecutive fields of view was employed to capture the whole section and all sections from a mouse using ImagePro Plus software to drive the microscope. Both GFP/Iba1 (microglia) and RFP (monocyte) channels were recorded. The resulting images were then analyzed with ImageJ software (National Institutes of Health) to calculate (1) lesion cavity volume, (2) the volume of brain tissue (in 4 mm slab) occupied by microglial immunoreactivity, and (3) volume of brain tissue occupied by monocyte immunoreactivity. Initially, each slice was manually outlined; for slices with a TBI-induced cavity, the “whole slice” outline was generated based on where the tissue would be if it were not missing. Then, the cavity, the region around the cavity with increased GFP/Iba1 immunoreactivity, and the region around the cavity with increased RFP immunoreactivity were manually outlined for each brain section. Finally, all sections (~22) for an animal were multiplied by the distance between sections (150 μm) and summed up to obtain the corresponding volumes (cavity, microglia, and monocyte immunoreactivity) as a percentage of the brain volume analyzed (4 mm thickness).

APP-stained sections were also imaged with tiling but in brightfield mode. To quantify axonal damage, the corpus callosum in the ipsilateral side to the injury was manually outlined in ImageJ and the percentage of the corpus callosum with APP immunoreactivity above threshold was calculated. Two sections were stained for each animal and averaged after quantification.

AT8-stained sections were imaged on a fluorescent Leica DM4000 microscope equipped with a QImaging Retiga EXi FAST 1394 MONO camera. Images were acquired at 10–20× magnification with a Cy5 filter cube (band-pass excitation 620/60 nm, band-pass emission 700/75 nm filters, 660 nm dichroic mirror). The same fields were also acquired through a DAPI filter cube (band-pass 360/40 nm excitation, band-pass 470/40 emission, 400 nm dichroic mirror) to localize cell nuclei. Images were taken of the ipsilateral cortex (close to the injury cavity), the underlying ipsilateral hippocampus and the contralateral cortex and hippocampus.

### Western blot and pTau quantification

pTau levels after TBI were quantified by Western blot. TBI and sham mice were anesthetized with ketamine/xylazine at 3 dpi and perfused with ice-cold PBS. Naïve mice that did not undergo any surgery were used as a control for the effect of surgery itself; naïve mice were simply anesthetized and perfused. The brains were isolated, the ipsilateral and contralateral hemispheres were separated (right and left for naïve mice), and the cortices and hippocampi were microdissected from each hemisphere. Tissues were homogenized in Tissue Protein Extraction Reagent (Pierce #78150) containing 1 % each of protease and phosphatase inhibitors (Sigma #P8340 and #P5726, respectively).

Fifty nanograms of total protein were separated on 4–12 % bis-tris gels (Invitrogen #NP0322) and transferred to 0.45 μm PVDF membranes (Millipore #IPFL10100). Separate membranes were run for the detection of phosphorylated tau (with mouse anti-AT8 antibody, 1:5000 dilution) and total tau (mouse anti-Tau5 antibody, Invitrogen #ahb0042. 1:5000). After running, the membranes were activated in methanol, blocked, and incubated in primary antibodies overnight at 4 °C. GAPDH (Trevigen #2275-PC-100, 1:10,000) was used as loading control on all membranes. Proteins were detected with goat anti-mouse IRDye 800CW (for AT8 or Tau5, Li-Cor #926-68171) and goat anti-rabbit IRDye 680RD (for GAPDH, Li-Cor #827-08364) fluorescent antibodies. Images of the developed membranes were acquired on a Li-Cor Odyssey CLx imager.

To quantify the relative levels of pTau across samples, the channels for tau and GAPDH were split and analyzed separately with the ImageJ Gel tool. Both AT8 and Tau5 signals were first normalized to GAPDH to ensure equal sample loading. Then, the relative AT8 signal was normalized to total tau. There were no significant differences in total tau between samples after TBI (data not shown).

### Statistical analysis

Data points on the graphs represent individual mice. The average and standard error of the mean are also shown. The effects of genotype and injury intensity were examined by two-way analysis of variance (ANOVA); statistical differences between groups were determined with Tukey’s *post hoc* tests. All significant values in *post hoc* tests were adjusted for multiple comparisons. Groups were considered to be statistically different if *p* < 0.05 for both factor effect (in ANOVA) and *post hoc* tests.

## Results

### Preventing CCR2^+^ monocyte infiltration after TBI reduces lesion pathology

The inflammatory reaction is a common feature of TBI. Here, we addressed how modulating monocytic inflammation might affect pathological features of neurodegeneration. We altered the inflammatory reaction in two different ways: by inactivation of *Cx3cr1* (in *Cx3cr1*^*GFP*/*GFP*^ mice), which affects the activation of brain-resident microglia [29] and Ly6C^lo^ patrolling monocytes, or by inactivation of *Ccr2* (in *Ccr2*^*RFP*/*RFP*^ mice), which reduces the infiltration of Ly6C^hi^ inflammatory monocytes and a subset of T cells into the brain after TBI [25]. Intercrossing these two mouse strains allowed us to not only inactivate the genes of interest, but also directly visualize chemokine (C-X3-C motif) receptor 1 (CX3CR1)- and CCR2-positive cells in tissue sections [26]. CX3CR1^+^ patrolling monocytes and CCR2^+^ T cells represent only a small proportion of infiltrating cells after TBI compared to CX3CR1^+^ microglia and CCR2^+^ inflammatory monocytes, respectively (data not shown). Hence, we herein refer to GFP-positive cells as microglia and RFP-positive cells as infiltrating inflammatory monocytes.

**Fig. 1:**
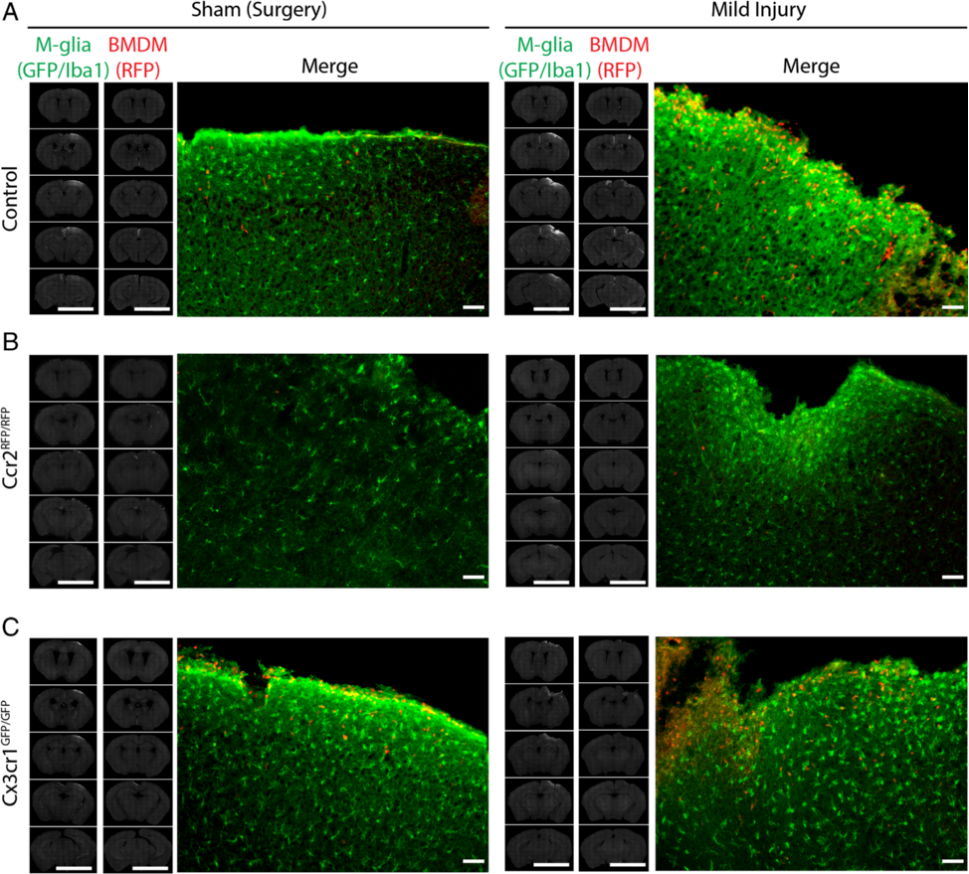
Modulation of the inflammatory reaction at 3 days after LFPI in CCR2 and CX3CR1 transgenic mice. Control *Ccr2* ^*RFP*/+^;*Cx3cr1* ^*GFP*/+^ (**a**), *Ccr2* ^*RFP*/*RFP*^ (**b**), and *Cx3cr1* ^*GFP*/*GFP*^ (**c**) mice were subjected to surgery (craniotomy; Sham mice) or mild injury, and brains were collected 3 days later. Serial sections were stained for GFP (**a**, **b** (Mild), and **c**) or Iba1 (**b** (Surgery)) to visualize microglia or RFP to visualize infiltrating bone marrow-derived monocytes (BMDM). Inflammatory cells are mostly restricted to the site of injury. *Scale bar* on images with whole slices, 1 cm. The brightness on the images was adjusted to allow an easier distinction of the brain sections from the background; all sections in a series were adjusted to the same brightness level. Magnified images show a close up view of microglial and monocyte distribution around the cavity size. Note the reduced number of RFP^+^ cells in *Ccr2* ^*RFP*/*RFP*^ mice. *Scale bar* for magnified images, 100 μm

*Ccr2*^*RFP*/*RFP*^, *Cx3cr1*^*GFP*/*GFP*^, and control double-heterozygous mice were subjected to mild LFPI, and the inflammatory reaction and cavity pathology were analyzed 3 dpi from brain sections spanning a 4-mm portion of the brain that encompassed the whole lesion area (Fig. 1). As expected, the injury induced microglial morphological transformation (seen as increased GFP/Iba1 signal) and infiltration of hematogenous monocytes (presence of RFP^+^ cells) at the site of damage in control double-heterozygous *Cx3cr1*^*GFP*/+^; *Ccr2*^*RFP*/+^ mice (Fig. 1a). The increased GFP signal and presence of RFP^+^ cells were mostly restricted to the site of tissue damage and brain cavitation. Moreover, the GFP^+^ cells had mainly ramified, bushy, morphology, suggesting that at this early time point, these cells were likely activated microglia rather than infiltrating CX3CR1^+^ monocytes. Interestingly, although care was taken not to disturb the dura mater and underlying brain during craniotomy, the “sham” surgery itself induced a low level of microglial activation and monocyte infiltration (Fig. 1a). Consistent with previous studies [23, 30], deletion of *Ccr2* prevented the infiltration of peripheral CCR2-positive cells into the brain after TBI as evident by the absence of RFP^+^ cell staining at the injury site (Fig. 1b). However, there was still prominent microglial reaction as monitored by GFP or Iba1 staining (Fig. 1b). In contrast, neither microglial activation nor monocyte infiltration in response to TBI appeared altered in *Cx3cr1*^*GFP*/*GFP*^ mice at 3 dpi (Fig. 1c).

**Fig. 2:**
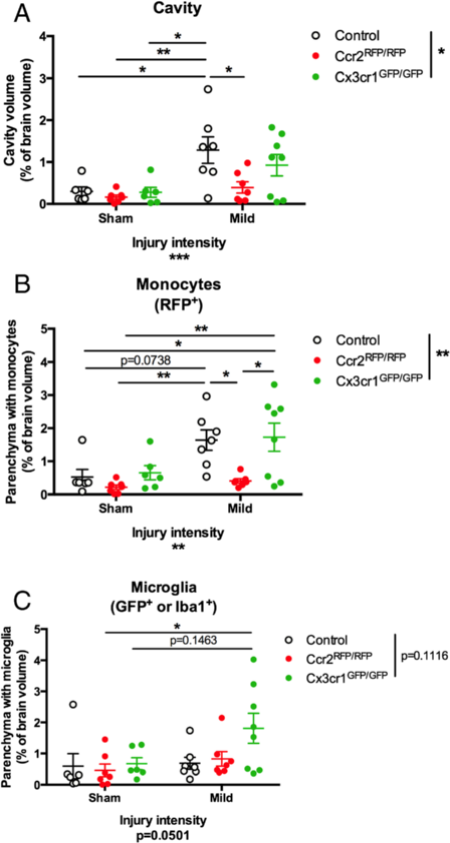
Quantification of cavity volume and inflammatory reaction after TBI. Serial sections were stained for GFP (or Iba1) to visualize microglia or RFP to visualize infiltrating bone marrow-derived monocytes (BMDM). Whole slices, lesion cavity, and area of the slices occupied by monocytes (RFP^+^ cells) or microglia (increased GFP or Iba1 immunoreactivity) were manually outlined to calculate their area; all sections were summed up to calculate volumes. Pathology was quantified as a percentage of the analyzed brain tissue (4 mm total thickness) that contained the lesion cavity (**a**), infiltrating monocytes (**b**), or increased microglial staining (**c**). Statistics: two-way ANOVA and Tukey’s *post hoc* test. Comparisons between groups are shown with *horizontal lines*; *vertical line* in figure legend indicates main effect of genotype. *p < 0.05; **p < 0.01; ***p < 0.001

We quantified the inflammatory reaction and the cavity pathology by calculating the percentage of the brain parenchyma (in a 4-mm brain portion around the injury) that was occupied by reactive microglia, infiltrating monocytes or size of tissue cavity (Fig. 2). Both injury and genotype had a significant effect on the cavity volume (Fig. 2a; two-way ANOVA, *p* < 0.001 for Injury and *p* < 0.05 for Genotype) and monocytic reaction (Fig. 2b; two-way ANOVA, *p* = 0.0010 for Injury and *p* < 0.01 for Genotype), but there was no significant change in the microglial reaction under any conditions examined (Fig. 2c; two-way ANOVA, *p* = 0.0501 for Injury and *p* = 0.1116 for Genotype). Consistent with the tissue staining (Fig. 1), *Ccr2* deletion resulted in a significant decrease in cavity volume (Fig. 2a; two-way ANOVA and Tukey’s *post hoc* test, *p* < 0.05 compared to heterozygous control) and portion of the brain parenchyma containing monocytes (Fig. 2b; two-way ANOVA and Tukey’s *post hoc* test, *p* < 0.05 compared to heterozygous control). Despite this, there was no change in the microglial reaction in *Ccr2*^*RFP*/*RFP*^ mice (Fig. 2c; two-way ANOVA and Tukey’s *post hoc* test, *p* = 0.9606).

Deletion of *Cx3cr1* did not affect the inflammatory reaction or cavity volume when comparing knockout to heterozygous mice (Fig. 2a-c; two-way ANOVA and Tukey’s *post hoc* test, *p* = 0.2205 for cavity volume, *p* = 0.0781 for monocytic reaction, and *p* = 0.1463 for microglial reaction). Because *Cx3cr1* deletion did not affect either the inflammatory reaction or cavity volume after mild injury here, we continued all subsequent studies only with *Ccr2*^*RFP*/*RFP*^ mice and appropriate controls.

### *Ccr2* deletion reduces TBI-induced axonal pathology

**Fig. 3:**
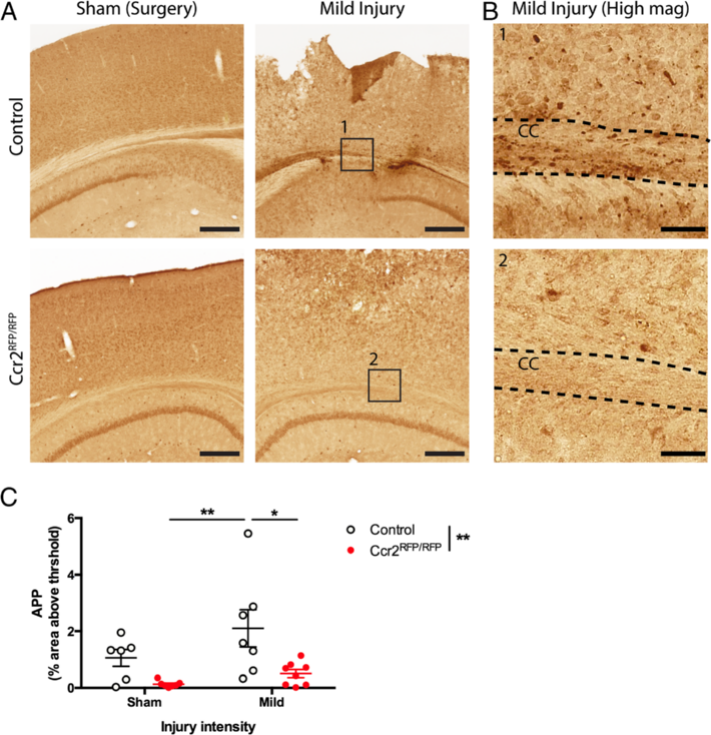
Effects of CCR2 signaling on axonal damage after TBI. Axonal pathology was evaluated at 3 dpi in control heterozygous and *Ccr2* ^*RFP*/*RFP*^ mice by APP staining. **a** The injury induces APP accumulation in axons of the ipsilateral side to the injury, particularly in the corpus callosum. *Ccr2* deletion decreases APP accumulation in axons. *Scale bar*, 500 μm. **b** Higher magnification images of the indicated regions (*boxes*) show that APP immunoreactivity aligns with axonal tracks in the corpus callosum (CC). *Scale bar*, 100 μm. **c** Quantification of axonal pathology as percent area of the corpus callosum with APP immunoreactivity above threshold. Statistics: two-way ANOVA and Tukey’s *post hoc* test. Comparisons between groups are shown with *horizontal lines*; *vertical line* in figure legend indicates main effect of genotype. *p < 0.05; **p < 0.01

After TBI, axonal pathology is characterized by APP-positive axonal swellings and axonal retraction bulbs indicating axotomy at sites remote to the injury [28]. In the present study, we detected increased APP immunoreactivity, especially in the corpus callosum underlying the injury site at 3 dpi (Fig. 3a). Examining the corpus callosum at higher magnification showed that many of the APP-positive structures seemed to align with the direction of axonal tracts (Fig. 3b). Deletion of *Ccr2* appeared to reduce APP immunoreactivity after TBI. Quantifying axonal pathology as the percentage of the corpus callosum with APP immunoreactivity confirmed reduced axonal pathology in *Ccr2*^*RFP*/*RFP*^ mice at 3 dpi compared to controls (Fig. 3c; two-way ANOVA and Tukey’s *post hoc* test, *p* < 0.05).

### *Ccr2* deletion alters tau phosphorylation and localization

**Fig. 4:**
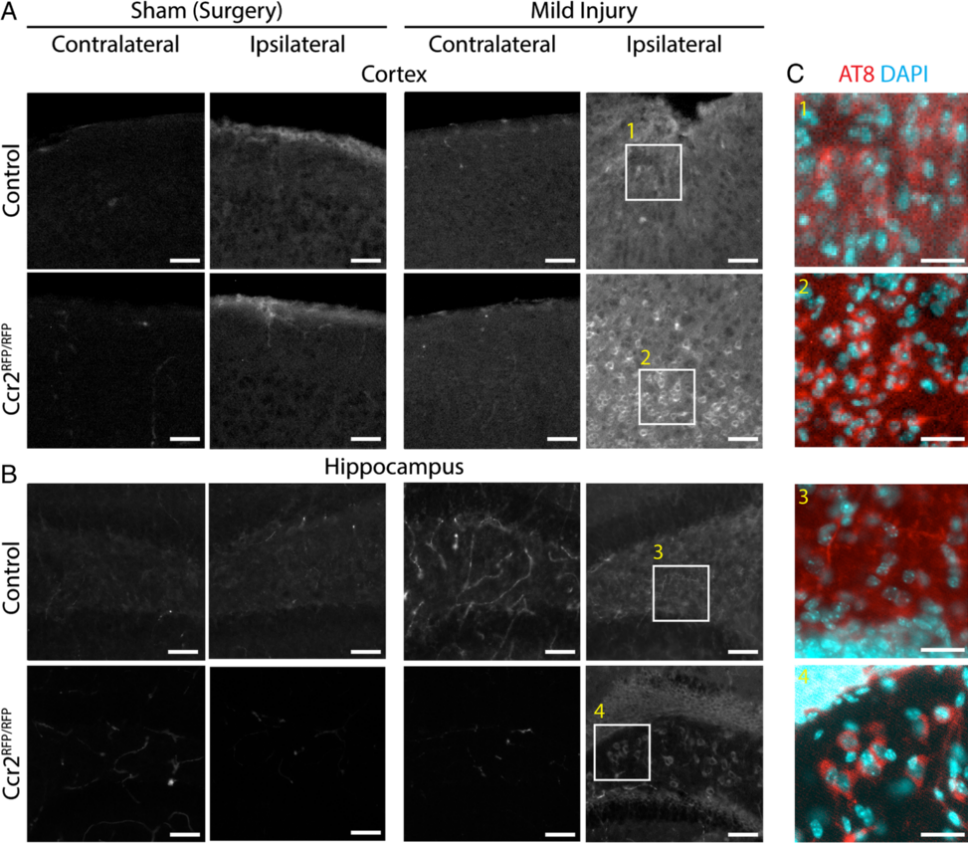
Assessment of tau phosphorylation and localization by tissue staining. Sections from control heterozygous and *Ccr2* ^*RFP*/*RFP*^ mice after surgery (Sham) or mild injury were stained with the AT8 pTau antibody. Images were taken from the cortex near the lesion cavity (**a**) and the underlying hippocampus (**b**) and the corresponding contralateral side. Injury induces increased pTau immunoreactivity as background neuropil staining in control mice and mislocalization to the cell body in *Ccr2* ^*RFP*/*RFP*^ mice. *Scale bar*, 100 μm. **c** *Insets* show pTau staining (AT8, *red*) in relation to DAPI-positive nuclei (*cyan*) for the indicated ipsilateral regions (*boxes*). *Scale bar*, 50 μm

Because inflammation has been shown to modulate tau pathology and *Ccr2* deletion reduces monocyte-mediated inflammation after TBI [10–12, 23, 30], we examined the effect of TBI on tau phosphorylation in *Ccr2*^*RFP*/*RFP*^ mice. We initially analyzed changes in tau phosphorylation by fluorescent immunohistochemistry to determine the localization of pTau (Fig. 4). Tissue staining for the AT8 pTau epitope showed increased staining at the site of injury in the cortex of both control heterozygous and *Ccr2*^*RFP*/*RFP*^ mice (Fig. 4a). While the pTau immunoreactivity at the lesion site appeared rather diffuse in heterozygous mice, pTau signal was mostly perinuclear in *Ccr2*^*RFP*/*RFP*^ mice (Fig. 4c). It should be noted that the physiological location of tau is in the axon; hence, both increased phosphorylation and mislocalization to the cell soma after TBI are abnormal.

Increased tau phosphorylation was also detected remote to the injury at 3 dpi, in the underlying hippocampus in both control and *Ccr2*^*RFP*/*RFP*^ mice (Fig. 4b). However, the pattern of pTau immunoreactivity appeared different in the two genotypes. As in the cortex, AT8 immunoreactivity in the hippocampus of heterozygous *Ccr2*^*RFP*/+^ TBI mice was mostly diffuse and located in the hilus of the dentate gyrus, outside the granule cell layer. In contrast, AT8 signal in the hippocampus of *Ccr2*^*RFP*/*RFP*^ mice was the highest around cell bodies in the granule cell layer of the dentate gyrus and hilus (Fig. 4b, c). Thus, TBI increases tau phosphorylation in both control and *Ccr2*^*RFP*/*RFP*^ mice, but only *Ccr2*^*RFP*/*RFP*^ mice show clear pTau translocation to the cell soma.

### Both injury and *Ccr2* genotype affect pTau levels by Western blot

**Fig. 5:**
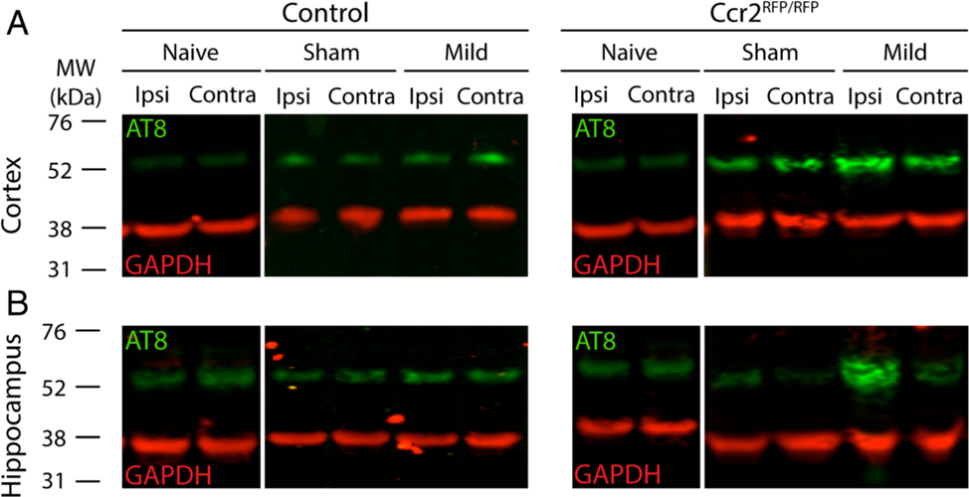
Assessment of tau phosphorylation by Western blot. Protein lysates from the cortex (**a**) or hippocampus (**b**) of treatment-naïve, sham (surgery only), or mild injured control or *Ccr2* ^*RFP*/*RFP*^ mice were probed for pTau (AT8 antibody, *green*). GAPDH was used as loading control (*red*). Mild injury induced a visible increase on the ipsilateral side of the cortex and hippocampus in *Ccr2* ^*RFP*/*RFP*^ mice

In order to quantify the changes in pTau after TBI, we performed Western blot analysis for the AT8 pTau epitope in microdissected cortical or hippocampal lysates. Because the hub-placement surgery itself influenced the inflammatory reaction (Fig. 1) and pTau immunoreactivity (Fig. 4a, compare sham ipsilateral and contralateral staining), we also included treatment-naïve mice that did not undergo surgery but received ketamine/xylazine anesthesia. Interestingly, there was not an injury- and side-dependent increase in pTau levels in the cortex at 3 dpi (Fig. 5a). Yet, mice with both sham surgery and mild LFPI exhibited higher AT8 pTau levels than naïve mice in a side-independent manner (Fig. 5a). The levels of pTau were also modulated differentially in the hippocampus. While hub-placement surgery did not affect pTau in control mice, they increased pTau in *Ccr2*^*RFP*/*RFP*^ mice in a “dose”- and side-dependent manner. That is, even surgery itself led to higher pTau on the ipsilateral side compared to naïve mice, and the higher “dose”—mild injury—led to an even larger increase in pTau (Fig. 5b).

**Fig. 6:**
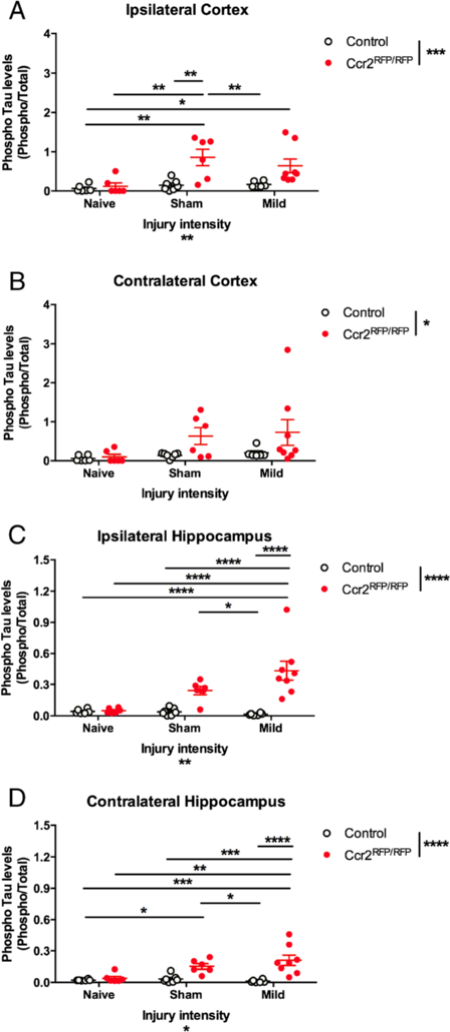
Quantification of pTau Western blots. The levels of pTau (AT8 antibody) were normalized to the levels of total tau (Tau5 antibody). **a**, **b** *Ccr2* genotype significantly affects pTau levels in both the ipsilateral (**a**) and contralateral (**b**) cortex, but pTau levels are elevated after experimental manipulation (surgery or mild injury) only on the ipsilateral side. **c**, **d** pTau levels in the hippocampus are increased on both the ipsilateral (**c**) and contralateral (**d**) side in injury- and genotype-dependent manner. As in the cortex, sham surgery itself elevates pTau levels in the hippocampus. Statistics: two-way ANOVA and Tukey’s *post hoc* test. Comparisons between groups are shown with *horizontal lines*; *vertical line* in figure legend indicates main effect of genotype. *p < 0.05; **p < 0.01; ***p < 0.001; ****p < 0.0001

We quantified pTau levels on the ipsilateral and contralateral sides separately in order to evaluate the effects of injury intensity and genotype (Fig. 6). In the ipsilateral cortex, both injury and genotype had a significant effect on pTau levels (Fig. 6a; two-way ANOVA, *p* < 0.01 for Injury and *p* < 0.001 for Genotype). Overall, *Ccr2* deletion resulted in higher pTau levels after experimental manipulation of the mice. There was a significant increase in pTau in *Ccr2*^*RFP*/*RFP*^ mice in sham (surgery) mice compared to naïve mice (two-way ANOVA and Tukey’s *post hoc* test, *p* < 0.01) but not an additional increase after mild injury (Fig. 6a). There was a significant difference between control and *Ccr2*^*RFP*/*RFP*^ mice only for the sham surgery groups (Fig. 6a; two-way ANOVA and Tukey’s *post hoc* test, *p* < 0.01). Despite the change in pTau immunoreactivity in tissue staining (Fig. 4), there was not a significant difference after injury in pTau levels in control heterozygous mice (Fig. 6a). *Ccr2*^*RFP*/*RFP*^ mice were also significantly different from control mice on the contralateral side of the cortex (two-way ANOVA, *p* < 0.05), but there were no significant inter-group changes (Fig. 6b).

Consistent with tissue staining (Fig. 4b), we detected robust changes in pTau levels in the hippocampus on both the ipsilateral and contralateral sides (Fig. 6c, d), with both injury and genotype having significant effects (two-way ANOVA, *p* < 0.0001 for Genotype for both the ipsilateral and contralateral sides, *p* < 0.01 and *p* < 0.05 for Injury intensity for the ipsilateral and contralateral sides, respectively). There was a significant difference in pTau levels after mild injury between control heterozygous and *Ccr2*^*RFP*/*RFP*^ mice (two-way ANOVA and Tukey’s *post hoc* test, *p* < 0.0001 for both sides). As in the cortex, the sham surgery induced an increase in pTau in *Ccr2*^*RFP*/*RFP*^ mice compared to naïve mice that was not subsequently increased by mild injury (Fig. 6c, d; two-way ANOVA and Tukey’s *post hoc* test, *p* < 0.0001 and *p* < 0.01 comparing naïve and mild injured *Ccr2*^*RFP*/*RFP*^ mice on the ipsilateral and contralateral sides, respectively). Experimental manipulation or surgery did not significantly affect pTau levels in control heterozygous mice. Thus, *Ccr2* deletion resulted in increased pTau levels on both the ipsilateral and contralateral sides at 3 days after injury or sham surgery.

## Discussion

The inflammation that develops after TBI has the potential to influence subsequent neuronal pathology [14, 18]; preventing or mitigating aspects of inflammation could help prevent secondary tissue damage and promote recovery [17]. Because inflammation is also a feature of many neurodegenerative conditions, we wanted to examine if modulating inflammation will also affect aspects of neurodegeneration such as altered Tau phosphorylation. We initially examined two models of altered inflammation: deletion of the chemokine receptor CX3CR1, which primarily affects microglia in the brain, and deletion of the chemokine receptor CCR2, which primarily affects bone marrow-derived monocytes and their extravasation to sites of damage [25, 29]. Only reduced monocyte infiltration through *Ccr2* deletion reduced cavity volume and axonal pathology (Figs. 1, 2, and 3), which led us to examine its effects on tau phosphorylation. Surprisingly, despite the reduced accumulation of monocytes at 3 dpi, *Ccr2*^*RFP*/*RFP*^ mice showed increased pTau levels by both tissue staining and Western blot analyses (Figs. 4, 5, and 6). Moreover, pTau was translocated from the axon to the cell body in both the cortex and hippocampus of *Ccr2*^*RFP*/*RFP*^ mice (Fig. 4). The pathology seen in TBI mice here is reminiscent to the neurofibrillary tangles seen in human tauopathies [31] and in patients with CTE or after a single TBI event [3, 32, 33].

### Modulation of inflammation in *Cx3cr1*^*GFP/GFP*^ and *Ccr2*^*RFP/RFP*^ mice

The *Cx3cr1*^*GFP*/*GFP*^ mice that we employed here are commonly used to study and visualize microglia in the brain [29, 34]. Indeed, parenchymal microglia are the predominant cell type expressing CX3CR1—and GFP—in the brain as peripheral CX3CR1-positive cells do not cross the intact blood–brain barrier (BBB) in physiological conditions. However, disruption of the BBB in TBI will allow peripheral CX3CR1^+^ cells to enter the brain. In a dorsal column crush model of spinal cord injury, CX3CR1^+^ “patrolling” monocytes mediate axonal pathology [35]. In our studies, the great majority of GFP^+^ cells after TBI had ramified or bushy morphology (Fig. 1), suggesting that they are microglia. Yet, additional studies are necessary to establish the ontology of all GFP^+^ cells after TBI.

Delivery of mild TBI to *Cx3cr1*^*GFP*/*GFP*^ mice did not significantly affect any of the pathological signs we examined: microglial activation, recruitment of CCR2^+^ cells, or lesion cavity, which was in contrast to the clear effect of *Ccr2* deletion (Figs. 1 and 2). Thus, we focused on CCR2 signaling rather than CX3CR1 signaling in subsequent analyses. It should be noted that microglial CX3CR1 signaling has been shown to affect tau phosphorylation in models of tauopathy. Specifically, deletion of *Cx3cr1* or its ligand *Cx3cl1* results in increased tau phosphorylation in mice overexpressing human tau or the APPPS1 model of AD [11, 36, 37]. Hence, it is plausible that *Cx3cr1* deletion might lead to changes in tau phosphorylation in our mild TBI model, but this question remains to be addressed.

As with *Cx3cr1*^*GFP*/*GFP*^ mice, *Ccr2*^*RFP*/*RFP*^ mice are simplistically used to study responses by inflammatory CD45^hi^Ly6C^hi^ monocytes [25, 26]. However, subsets of T cells, NK cells, and Ly6C^lo^ monocytes express CCR2 or the RFP reporter in some conditions [26]. Although the frequency of these other CCR2/RFP^+^ cells is significantly lower than the frequency of CCR2^+^ inflammatory monocytes after TBI [24], we cannot rule out that these cell types contribute to the pathology. Thus, additional studies will be necessary to carefully phenotype the different lymphoid populations that respond to TBI and their role in TBI outcomes.

### Tau phosphorylation after TBI

There is ample evidence that phosphorylated, aggregated tau is present in the brain of humans that had suffered from various tauopathies, including CTE, AD, frontotemporal dementia, Pick’s disease, and others [31]. In the context of TBI, pTau has been observed in numerous individuals who had experienced traumatic events ranging from repetitive sports-related injuries to isolated head traumas (for examples, see 32, 3). Yet, there are only a handful of studies that have examined tau phosphorylation in animal models of TBI. In two different transgenic tau-overexpressing animals, TBI accelerates tau pathology [38, 39]. Using wild type mice, Genis et al. [40] and Iliff et al. [41] show that TBI can cause tau hyperphosphorylation in the absence of mutated transgene expression. However, although Genis et al. [40] suggest that tau phosphorylation is transient, peaking at 4 h, Iliff et al. [41] demonstrate increased levels of pTau in the brain even at 28 days after injury. Similar to our study, they found that increased tau phosphorylation is not restricted to the injured ipsilateral side, but is also detectable to the contralateral side.

Another noteworthy observation from our study is that the surgery that is required to prepare mice for fluid percussion injury is sufficient to increase pTau levels when compared to treatment-naïve mice. Furthermore, certain types of anesthetics quickly increase the levels of pTau in the brain [42–44]. In our study, all groups received anesthesia, including the treatment-naïve mice. Thus, the effects of anesthesia alone are not sufficient to explain the increase of pTau after surgery and injury. Yet, these findings suggest that tau phosphorylation may represent an immediate physiological response to brain disturbances or a biomarker of altered brain homeostasis. It is worth investigating if tau becomes phosphorylated in other conditions in which brain function is impaired, such as stroke, epilepsy, etc. Moreover, although pTau is more prone to aggregation [9], it remains to be determined if it serves physiological functions that may be aimed at returning the brain to homeostasis in the acute time scales.

We were not able to detect a significant increase in pTau levels after mild injury compared to the surgery itself. One possibility for this is that the surgery and *Ccr2*^*RFP*/*RFP*^ genotype synergized to produce a strong pTau signal, and the deletion of *Ccr2* itself sensitizes the mice to anesthesia- or surgery-mediated effects on pTau. Then, the delivery of an additional injury with mild intensity may not be sufficient to substantially increase pTau levels (Fig. 6). However, despite the lack of protein increase by Western blot, the injury does induce pTau translocation from the axon to the cell body (Fig. 4). As a result, surgery (or other brain disturbances) and traumatic injury may play distinct, sequential roles to lead to frank tau pathology.

### Ccr2 modulation in TBI

Preventing monocyte accumulation in the brain has become a promising therapeutic approach to treat TBI and prevent the development of secondary pathologies. Deletion of *Ccl2*, a ligand for CCR2, *Ccr2* itself or antagonism of CCR2 with small molecules have all been shown to be beneficial after TBI [23, 30, 45–47]. Importantly, interfering with CCR2 signaling results in improved cognitive function for up to 4 weeks after the injury.

Yet, here we show that *Ccr2* deletion also leads to increased phosphorylation of tau protein—an event that is commonly associated with loss of physiological function and gain of pathological function by tau in a variety of neurodegenerative diseases [31]. In many of these conditions, tau is found in hyperphosphorylated form in the cell body of neurons, similar to what we observed here after TBI in *Ccr2*^*RFP*/*RFP*^ mice. This raises the question whether interfering with CCR2 signaling might have unexpected consequences of promoting the development of pathologies.

There are still important questions that need to be addressed about the role of CCR2 signaling in tau pathology after TBI. First, are the effects of *Ccr2* deletion on tau phosphorylation long lasting or transient? Second, are other disease-associated epitopes of pTau, such as AT180, PHF-1, or CP13, affected? The work by Iliff et al. [41] suggests that tau phosphorylation could persist for at least 28 days after TBI and affect a variety of pTau epitopes. Finally, how can the absence of a peripheral cell type, which is normally not found in the brain, lead to increased tau phosphorylation? One possible answer to this last question is that the infiltrating monocytes modulate the function of resident brain cells such as neurons, astrocytes, and microglia in the context of TBI; in the absence of infiltrating monocytes, the resident cells may release factors that in turn regulate tau phosphorylation. Indeed, we did not detect a reduction in the brain parenchyma occupied by activated microglia in *Ccr2*^*RFP*/*RFP*^ mice (Figs. 1 and 2). Whether these microglia are functionally and molecularly different from microglia in control mice after TBI remains to be determined. Similarly, whether the function of other brain-resident cell types is affected by the absence of CCR2^+^ cells in TBI still needs to be examined.

## Conclusions

Here we show an unexpected outcome of inhibiting monocyte infiltration into the brain after TBI in *Ccr2*^*RFP*/*RFP*^ mice. Although *Ccr2* deletion is protective in terms of lesion volume, axonal pathology, and subsequent behavioral outcomes, it leads to increased phosphorylation and mislocalization of tau protein. The latter has been associated with neurodegenerative diseases and should be carefully considered if strategies aimed at CCR2 signaling continue to be pursued for therapeutic purposes in TBI and other conditions.

## Abbreviations

AD: Alzheimer’s disease
ANOVA: analysis of variance
APP: amyloid precursor protein
BBB: blood–brain barrier
CCL2: chemokine (C-C motif) ligand 2
CCR2: chemokine (C-C motif) receptor 2
CNS: central nervous system
CX3CR1: chemokine (C-X3-C motif) receptor 1
CTE: chronic traumatic encephalopathy
dpi: days post injury
GFP: green fluorescent protein
LFPI: lateral fluid percussion injury
MAPT: microtubule-associated protein tau
NGS: normal goat serum
PBS: phosphate-buffered saline
PFA: paraformaldehyde
pTau: (hyper)phosphorylated tau
RFP: red fluorescent protein
RT: room temperature
TBI: traumatic brain injury
